# Thought disorder is correlated with atypical spoken binomial orderings

**DOI:** 10.1038/s41537-022-00238-8

**Published:** 2022-03-18

**Authors:** Michael Murphy, Dost Öngür

**Affiliations:** 1 Harvard Medical School, Boston, MA USA; 2 McLean Hospital, Belmont, MA USA

**Keywords:** Psychosis, Human behaviour, Schizophrenia

## Abstract

Thought disorder may be associated with subtle language abnormalities. Binomials are pairs of words of the same grammatical type that are joined by a conjunction that often have a preferred order (for example, “up and down” is more common than “down and up”). We analyzed speech transcripts from patients with first-episode psychosis and found that atypical ordering of binomial pairs was associated with thought disorder but not with other psychosis symptoms. These results illustrate the potential to generate objective, quantifiable measures of disorganized speech.

## Main text

Linguistic and semantic properties of spoken language are abnormal in patients with psychotic disorders. Thought disorder is associated with quantifiable and objective patterns of patient speech. Identifying speech patterns that are correlated with thought disorder may be particularly important for first-episode populations as thought disorder is highly linked with treatment failure in this population^1^. Neuroimaging studies suggest that patients with thought disorder have specific neurological deficits that may require targeted treatments^2^. Patients with disorganized speech have diminished syntactic complexity and aberrant use of acausal connective words such as “first”^3,4^. Other measures may even be able to detect subclinical thought disorder symptoms. Tang et al. used Bidirectional Encoder Representations from Transformers (BERT) to analyze speech from patients with psychosis who had low levels of clinician-assessed formal thought disorder^5^. They found that the embedding distance from an interview prompt increased across sentences in patients but decreased in controls.

Binomial pairs often have a preferred ordering, which can be demonstrated using the Google Books ngram database^6,7^. Speaker selection of the preferred ordering requires a complex implicit weighting of phonetic, semantic, and statistical factors, such as word length, word frequency, and temporal sequencing as well as recall of which ordering is more frequently encountered^6^. This makes binomial ordering a subtle and naturalistic probe of cognitive functioning. In people with disorganized speech, failure to inhibit distractors and aberrant semantic priming combine to create altered and inappropriate context for words^8^. We hypothesized that the same processes that lead to disorganized speech disrupt the selection of binomial orderings and that this manifests as an increase in less common orderings for binomials.

**Fig. 1:**
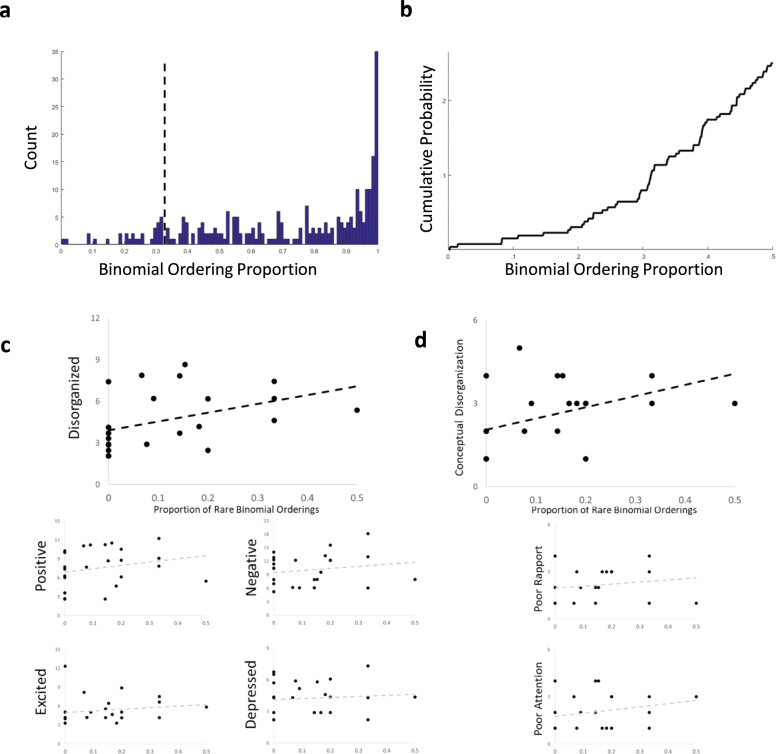
Rare binomials are associated with thought disorder. **a** A histogram of the binomial ordering preference for all binomials across all subjects. The dotted line is at 0.33 and all binomials to the left of this line were considered to be rare binomials. **b** The cumulative probability of all binomial ordering preferences (up to 0.5) across all subjects. **c** Scatter plots between number of rare binomials, normalized by total number of binomials per transcript, and the factor scores. Only the black trendline (Disorganized Factor) is a statistically significant association. **d** Scatter plots showing the relationships between normalized rare binomial count and the three component PANSS items of the Disorganized Factor score. Only the Conceptual Disorganization item shows a statistically significant relationship.

We analyzed transcripts from Structured Clinical Interview for the Positive and Negative Symptom Scale (SCI-PANSS) interviews of 28 patients with first-episode psychosis and compared disorganization factor scores to binomial ordering statistics. Participants produced a highly variable number of words (mean = 3876.1 with standard deviation = 2714.1) and binomials (mean = 8.5 with standard deviation of 8.1). Binomial count was highly correlated with total number of words spoken (Pearson’s r = 0.88). We used the histogram of all binomial ordering preferences across participants and the cumulative probability across likelihoods to select a threshold for considering a binomial ordering to be “rare” (Fig. 1a). Any ordering that occurred less than 0.33 (that is, was outscored 2 to 1) in the corpus was considered a rare binomial ordering. We expected that participants who used more binomials in their speech were more likely to produce rare binomials and therefore, for each participant, we divided the number of rare binomial orderings by the total number of binomials. The normalized proportion of rare binomial orderings was not statistically significantly different in men and women (unpaired *t*-test, *p* = 0.19).

Disorganization factor score was statistically significantly correlated with the proportion of rare binomial orderings (Fig. 1b; Spearman’s r = 0.52, *p*_adjusted_ = 0.046). No other factor score was associated with proportion of rare binomial orderings (all *p*_adjusted_ > 0.33. The Disorganization factor score is a weighted sum of three PANSS items: Conceptual Disorganization, Poor Rapport, and Poor Attention^9^. We then tested whether any of these measures were correlated with proportion of rare binomial orderings. We found that only the Conceptual Disorganization item was correlated with rare binomial orderings (Spearman’s r = 0.54, *p*_adjusted_ = 0.018; the other components had *p*_adjusted_ > 0.36).

We found that disorganized speech is accompanied by grammatically correct but unusual ordering of binomial pairs. This may arise from impaired cognitive control processes that are unable to reliably produce the preferred binomial ordering and/or impaired recall of the more frequently encountered ordering. Our results are consistent with recent work showing that disorganized speech is associated with idiosyncratic use of function words including conjunctions^10,11^.

This study has several limitations. The sample size was limited and there was no healthy control comparison group. The participants in this study were assessed using the PANSS, a general psychosis scale, rather than a more fine-grained thought-disorder-specific scale. There may be subtypes of thought disorder, such as positive thought disorder, that are associated with atypical binomial orderings and other subtypes, such as negative thought disorder, that are not^8^. Future work should use larger sample sizes, incorporate scales that can disaggregate thought disorder subtypes, and include directly probing participants ability to order novel binomials.

## Methods

### Participants and procedure

All study procedures were approved by the Institutional Review Board of Partners Healthcare/Mass General Brigham. Twenty-eight people with first-episode psychosis (within 3 years of initial diagnosis) were recruited from inpatient units and outpatient clinics at McLean Hospital. Exclusion criteria were limited to a history of head injury, neurological disorders, prior electroconvulsive therapy, and active major medical illness. Participants provided written informed consent. Demographic information for the included participants can be found in Table 1. Participants completed a SCI-PANSS interview, which was scored offline^12^. Factor scores (Positive, Negative, Disorganized, Excited, Depressed) were weighted sums of PANSS items with weights taken from a validated five-factor model^9^.

**Table 1:** Demographic features of the participants.

	Participants
*n*	28
Age (years)	22.5 ± 2.9
Sex (male/female)	17/11
Chlorpromazine (CPZ) equivalents (mg)	249.7 ± 178.0
Dx	
SZ/SZA	9
BP	15
Other	4
PANSS-positive factor	7.2 ± 3.2
PANSS-negative factor	10.5 ± 3.6
PANSS-disorganized factor	4.6 ± 2.1
PANSS-excited factor	5.5 ± 2.3
PANSS-depressed factor	4.2 ± 1.6

Values are mean ± standard deviation.

### Binomials and statistics

We identified a list of binomials linked with the word “and” from each interview transcript. Three participants used fewer than three binomials and were excluded from further analysis. For each binomial, we generated the reverse binomial, for example, for the binomial “write and paint” we generated the binomial “paint and write”. We then used the ngramr package in R to query the Google N-gram database for works published in English between 2010 and 2019^7,13,14^. For each binomial, we calculated occurrences of the true binomial divided by the sum of the occurrences of the true binomial and the reversed binomial (the “binomial ordering proportion”). For some binomials, the reverse ordering never occurred. These binomials were not included in the analysis. Spearman correlation was used to measure the relationships between clinical features and linguistic measures. Adjusted *p*-values were Bonferroni-corrected.

## Data Availability

Binomial lists and symptom scoring data are available from the corresponding author upon reasonable request.
